# A dual-slice *k-t* approach for highly accelerated flow MRI

**DOI:** 10.1186/1532-429X-14-S1-W47

**Published:** 2012-02-01

**Authors:** Daniel Giese, Tobias Schaeffter, Sebastian Kozerke

**Affiliations:** 1 Institute for Biomedical Engineering, University and ETH Zurich, Zurich, Switzerland; 2 Division of Imaging Sciences and Biomedical Engineering, King’s College London, London, UK

## Summary

We propose the combination of *k-t* undersampling with a dual slice excitation approach. The dual slice excitation and reconstruction principle is incorporated into the *k-t* SENSE reconstruction framework, allowing a doubling of the net acceleration factor when acquiring two slices as compared to acquiring two separate undersampled slices.

## Background

Phase contrast MRI has limited practical value in clinical applications due to its inherently long scan times[Kilner,JCMR'07]. Parallel imaging[Pruessmann,MRM'01] and spatio-temporal constrained reconstruction techniques[Tsao,MRM'03] have been shown beneficial[Baltes,MRM'05]. Dual slice approaches have shown advantages as compared to standard SENSE when multiple and sufficiently separated slices are acquired[Breuer,MRM'10]. We present a combination of dual slice excitation with undersampled flow imaging by modifying the *k-t* SENSE reconstruction framework to include sensitivity information in through-slice direction.

## Methods

**Figure 1:**
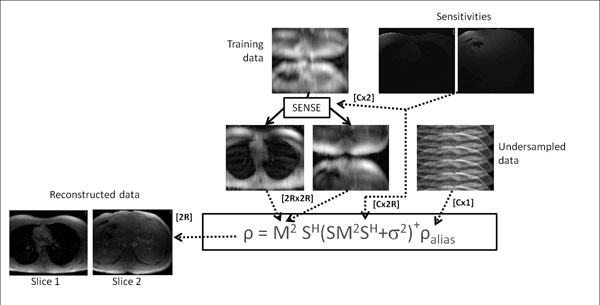
Reconstruction framework for dual-slice *k-t* imaging. The training data is unfolded prior to reconstruction yielding the training matrix term (**M**) in the reconstruction. Undersampled data is unfolded point-wise in *x-f* space using a similar approach as in single slice *k-t* SENSE, however solving for two slices simultaneously. **S** is the encoding matrix (including sensitivities), **ρ** the unfolded, **ρ_alias_** the folded pixels and **σ^2^** the noise covariance matrix. The square brackets show the variables dimensions per unfolded pixels (**R**: undersampling factor, **C**: number of coils).

The dual slice excitation was performed using a cosine modulated sinc pulse. A FOV/2 shift of one slice in phase encoding direction was achieved by alternating the phase of the RF pulse along the phase encoding direction. Dual slice *k-t* undersampled data was acquired in a healthy volunteer in a single breathhold using a 32 channel coil array. The transversal slices were separated by 120cm, one was placed at the level of the pulmonary artery, the other one at the level of the liver. Slices were flow encoded with a velocity encoding of 200cm/s. Prior to reconstruction, the training data was SENSE-unfolded. The *k-t* unfolding was modified and expanded by incorporating the dimension of the two acquired slices resulting in a single inversion process with doubled matrix size as compared to single slice *k-t* reconstruction (Figure 1). A nominal acceleration factor of R=5 was chosen with 11 training profiles (net acceleration factor of 3.6 per slice totaling 7.2 for the dual slice acquisition). For comparison, reference fully sampled as well as undersampled acquisitions of each seperate slice were acquired. Root mean squared errors (RMSE) of the flow profiles (in ml/s and % of the maximum flow) were calculated for dual slice *k-t* SENSE and single slice *k-t* SENSE.

## Results

**Figure 2:**
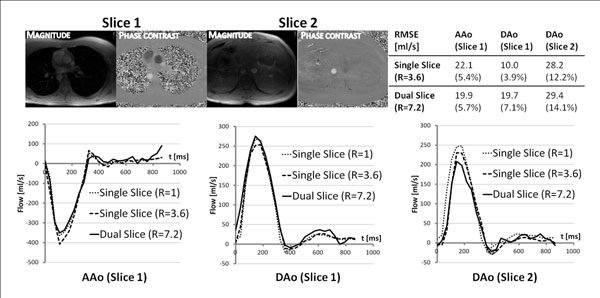
Initial results of dual-slice-*k-t* reconstructions. The images show the two simultaneously acquired spatio-temporally undersampled slices unfolded using the proposed method. Magnitude and phase contrast images are shown for a single heart phase. Flow curves through the ascending aorta (AAo) in slice 1 and the descending aorta (DAo) in slices 1 and 2 are shown. The fully sampled (R=1), the single slice *k-t* SENSE accelerated (R=3.6) and the dual slice (R=7.2) flow curves are shown. Root mean squared errors (RMSE) with respect to the reference data are shown for the accelerated techniques in ml/s as well as in percentage of the maximum flow.

Figure 2 shows *k-t* dual-slice unfolded magnitude and phase images. Temporal behavior and flow quantification are demonstrated by flow curves of the ascending aorta in the upper slice and the descending aorta in both slices. Reference flow curves along with single slice *k-t* SENSE and the proposed dual slice *k-t* approach are plotted showing similar temporal behaviors. RMSE with respect to fully sampled data confirm the agreement between flow curves.

## Conclusions

We have presented a reconstruction framework allowing reconstruction of two simultaneously acquired spatio-temporally undersampled phase-contrast slices. Initial results show good agreement between reference, fully sampled, single slice undersampled *k-t* SENSE and the proposed dual slice accelerated acquisitions. Slight variations between all three images are likely due to the differences in sequentially acquired data. Future work will include the adaptation into other spatio-temporal constrained reconstruction techniques (*k-t* PCA[Pedersen,MRM'09]) allowing for higher net acceleration factors as well as validation in volunteers and patients.

